# Surveillance for Foodborne Disease Outbreaks — United States, 2009–2010

**Published:** 2013-01-25

**Authors:** L. Hannah Gould, Elisabeth A. Mungai, Shacara D. Johnson, LaTonia C. Richardson, Ian T. Williams, Patricia M. Griffin, Dana J. Cole, Aron J. Hall

**Affiliations:** Div of Foodborne, Waterborne, and Environmental Diseases, National Center for Emerging and Zoonotic Infectious Diseases; Div of Viral Diseases, National Center for Immunization and Respiratory Diseases, CDC

Known pathogens cause an estimated 9.4 million foodborne illnesses annually in the United States (1). CDC collects data on foodborne disease outbreaks submitted by all states, the District of Columbia, and Puerto Rico through CDC’s Foodborne Disease Outbreak Surveillance System. Data reported for each outbreak include the number of illnesses, hospitalizations, and deaths; the etiologic agent; the implicated food vehicle; and other factors involved in food preparation and consumption. During 2009–2010, a total of 1,527 foodborne disease outbreaks (675 in 2009 and 852 in 2010) were reported, resulting in 29,444 cases of illness, 1,184 hospitalizations, and 23 deaths. Among the 790 outbreaks with a single laboratory-confirmed etiologic agent, norovirus was the most commonly reported, accounting for 42% of outbreaks. *Salmonella* was second, accounting for 30% of outbreaks. Among the 299 outbreaks attributed to a food composed of ingredients from one of 17 predefined, mutually exclusive food commodities (2), those most often implicated were beef (13%), dairy (12%), fish (12%), and poultry (11%). The commodities in the 299 outbreaks associated with the most illnesses were eggs (27% of illnesses), beef (11%), and poultry (10%). Public health, regulatory, and food industry professionals can use this information when creating targeted control strategies along the farm-to-table continuum for specific agents, specific foods, and specific pairs of agents and foods. This information also supports efforts to promote safe food-handling practices among food workers and the public.

CDC defines a foodborne disease outbreak as the occurrence of two or more similar illnesses resulting from ingestion of a common food. State, local, tribal, and territorial health department officials voluntarily submit reports of outbreaks investigated by their agency to the Foodborne Disease Outbreak Surveillance System on a standard, Internet-based form.* This report analyzes outbreaks that were reported by August 2, 2012, in which the first illness occurred during 2009–2010. Data reported for each outbreak include the number of illnesses, hospitalizations, and deaths; the etiologic agent (confirmed or suspected†); the implicated food vehicle; factors contributing to food contamination; and the settings of food preparation and consumption. Foods were assigned to one of 17 commodities§ if a single contaminated ingredient was identified or if all ingredients belonged to that commodity (2). Outbreaks identifying foods that could not be assigned to one of the 17 commodities, or for which the report contained insufficient information for commodity assignment, were not attributed to any commodity. Population-based outbreak reporting rates were calculated for each state using U.S. Census estimates of the 2009 and 2010 state populations.¶

Public health officials from all 50 states, the District of Columbia, and Puerto Rico reported 1,527 outbreaks, including 675 in 2009 and 852 in 2010. For the period 2009–2010, the median average annual rate of foodborne outbreaks among states was 3.2 per 1 million population (Figure).

A single confirmed or suspected etiologic agent was identified in 1,022 (67%) outbreaks (790 confirmed and 232 suspected) (Table 1). Among the 790 outbreaks with a single confirmed etiologic agent, bacteria caused 413 (52%) outbreaks, viruses caused 336 (42%), chemicals and toxins caused 39 (5%), and parasites caused 2 (0.2%). Norovirus was the most common cause of outbreaks and illnesses, accounting for 331 (42%) of the confirmed, single-etiology outbreaks and 7,332 (37%) illnesses. *Salmonella* was next, causing 234 (30%) of confirmed, single-etiology outbreaks and 7,039 (36%) illnesses. Among the 225 confirmed *Salmonella* outbreaks with a serotype reported, Enteritidis was the most common serotype with 76 outbreaks (34%). Shiga toxin–producing *Escherichia coli* (STEC) caused 58 confirmed, single-etiology outbreaks, of which 53 were caused by serogroup O157.

Of the 29,444 outbreak-related illnesses, 1,184 (4%) resulted in hospitalization. *Salmonella* caused the most outbreak-related hospitalizations with 583 (49%), followed by STEC with 190 (16%) and norovirus with 109 (9%). Outbreaks caused by *Listeria* resulted in the highest proportion of persons hospitalized (82%), followed by *Clostridium botulinum* (67%), and paralytic shellfish poisoning outbreaks (67%). Among the 23 deaths, 22 were attributed to bacterial etiologies (nine to *Listeria monocytogenes,* five *Salmonella*, four STEC O157, three *Clostridium perfringens,* and one *Shigella*), and one to norovirus.

A food vehicle was reported for 653 (43%) outbreaks; in 299 (46%) of these outbreaks the vehicle could be assigned to one of the 17 predefined commodities (Table 2). The commodities most commonly implicated were beef, with 39 outbreaks (13%), followed by dairy and fish with 37 (13%) each, and poultry with 33 (11%). Among the 36 dairy-associated outbreaks for which pasteurization information was reported, 26 (81%) involved unpasteurized products. The commodities associated with the most outbreak-related illnesses were eggs with 2,231 illnesses (27%), beef with 928 (11%), and poultry with 826 (10%). The pathogen-commodity pairs responsible for the most outbreaks were *Campylobacter* in unpasteurized dairy (17 outbreaks), *Salmonella* in eggs and STEC O157 in beef (15 each), ciguatoxin in fish (12), and scombroid toxin (histamine fish poisoning) in fish (10). The pathogen-commodity pairs responsible for the most outbreak-related illnesses were *Salmonella* in eggs (2,231 illnesses), *Salmonella* in sprouts (493), and *Salmonella* in vine-stalk vegetables** (422). The pathogen-commodity pairs responsible for the most hospitalizations were *Salmonella* in vine-stalk vegetables (88 hospitalizations), STEC O157 in beef (46), and *Salmonella* in sprouts (41). The pathogen-commodity pairs responsible for the most deaths were STEC O157 in beef (three deaths), and *Salmonella* in pork and *Listeria* in dairy (two each).

Thirty-eight multistate outbreaks were reported (16 in 2009 and 22 in 2010). Twenty-one were caused by *Salmonella,* 15 by STEC (13 O157, one O145, and one O26), and two by *Listeria*. The etiologic agent was isolated from an implicated food in 11 multistate outbreaks. Five of the multistate outbreaks were caused by *Salmonella* (in alfalfa sprouts [two outbreaks], ground turkey, shell eggs, and a frozen entrée [one each]). Six were caused by STEC (in ground beef [two outbreaks], unpasteurized Gouda cheese, multiple unpasteurized cheeses, hazelnuts, and cookie dough [one each]).

Among the 766 outbreaks with a known single setting where food was consumed, 48% were caused by food consumed in a restaurant or deli, and 21% were caused by food consumed in a private home. Forty-three outbreaks resulted in product recalls.†† The recalled foods were ground beef (eight outbreaks), sprouts (seven), cheese and cheese-containing products (six), oysters (five), raw milk (three), eggs (three), and salami (ground pepper), bison, sirloin steak, unpasteurized apple cider, cookie dough, frozen mamey fruit, hazelnuts, Romaine lettuce, ground turkey burger, tuna steak, and a frozen entrée (one each).

## Editorial Note

In 2009, the Foodborne Disease Outbreak Surveillance System transitioned to the use of a new reporting form and online data entry interface, the National Outbreak Reporting System (NORS). NORS receives reports of outbreaks of enteric disease transmitted through water, person-to-person contact, contact with animals, environmental contamination, and indeterminate means, as well as through food. Before 2009, only foodborne and waterborne outbreaks were reported to CDC. Following the transition to the new system, the number of foodborne disease outbreaks reported in 2009 and 2010 declined 32% compared with the mean of the preceding 5 years (4).

The decline in foodborne disease outbreak reporting was largely observed among norovirus outbreaks. Norovirus can be transmitted through a variety of routes, including direct contact between persons, through contact with contaminated surfaces, and ingestion of contaminated food or water (5,6). Distinguishing among these modes of transmission in an outbreak can be challenging; some outbreaks involve multiple transmission routes. The advent of NORS, which for the first time enables electronic reporting of nonfoodborne norovirus outbreaks, might have led to more appropriate classification of outbreaks previously reported as foodborne, resulting in fewer reports of foodborne norovirus outbreaks. Other possible explanations for the fewer foodborne disease outbreaks in 2009 and 2010 include resource limitations and competing priorities (e.g., the influenza A [H1N1] virus pandemic in 2009) for state epidemiologic and laboratory resources (7,8).

What is already known about this topic?

Surveillance for foodborne disease outbreaks can identify opportunities to prevent foodborne diseases, which cause millions of illnesses in the United States each year.

What is added by this report?

Among the 1,527 foodborne disease outbreaks reported in 2009 and 2010, most outbreak-associated illnesses were caused by norovirus or *Salmonella.* Among outbreaks in which both an etiologic agent and single-commodity food vehicle were identified, most outbreaks were attributed to *Campylobacter* in unpasteurized dairy products, *Salmonella* in eggs, and Shiga toxin–producing *Escherichia coli* O157 in beef. The pathogen-commodity pairs responsible for the most outbreak-related illnesses were *Salmonella* in eggs (2,231 illnesses), in sprouts (493), and in vine-stalk vegetables (422).

What are the implications for public health practice?

Public health, regulatory, and food industry professionals can use this information when creating targeted control strategies along the farm-to-table continuum for specific agents and foods, and specific pairs of agents and foods. This information also supports efforts to promote safe food-handling practices among food workers and the public.

For STEC O157 and *Salmonella* serotype Enteritidis, the number of outbreaks reported was not lower than previous years. For STEC O157, the 33 outbreaks in 2009 and 20 in 2010 exceeded the *Healthy People 2010* yearly target of 11, and for *Salmonella* serotype Enteritidis*,* the 39 outbreaks in 2009 and 37 outbreaks in 2010 exceeded the *Healthy People 2010* yearly target of 22 (9).

During 2009–2010, beef, dairy, fish, and poultry were associated with the largest number of foodborne disease outbreaks. During the preceding 11 years, beef, fish, and poultry were consistently among the commodities most commonly associated with outbreaks (4). The large number of outbreaks caused by unpasteurized dairy products is consistent with findings that more outbreaks occur in states that permit the sale of unpasteurized dairy products (10); 60% of states permit sales of raw milk in some form, according to a 2011 survey by the National Association of State Departments of Agriculture.§§

The findings in this report are subject to at least four limitations. First, only a small proportion of foodborne illnesses reported each year are identified as associated with outbreaks. The extent to which the distributions of food vehicles and settings implicated in foodborne disease outbreaks reflect the same vehicles and settings as sporadic foodborne illnesses is unknown (4). Similarly, not all outbreaks are identified, investigated, or reported. Second, many reported outbreaks had an unknown etiology, an unknown food vehicle, or both, and conclusions drawn from outbreaks with a confirmed or suspected etiology or food vehicle might not apply to outbreaks with an unknown etiology or food vehicle. Even when a food is identified, the point of contamination is not always known or reported. Third, CDC’s outbreak surveillance system is dynamic; agencies can submit new reports and can change or delete previous reports as new information becomes available. Therefore, the results of this analysis might differ from those published earlier or from future reports. Finally, because of changes in the surveillance system implemented in 2009, comparisons with preceding years should be made with caution.

Public health, regulatory, and food industry professionals use foodborne disease outbreak surveillance data to target prevention efforts related to pathogens and foods that cause foodborne disease outbreaks. Additional information on outbreaks and the Foodborne Outbreak Online Database are available at http://www.cdc.gov/outbreaknet/surveillance_data.html.

## Figures and Tables

**FIGURE:**
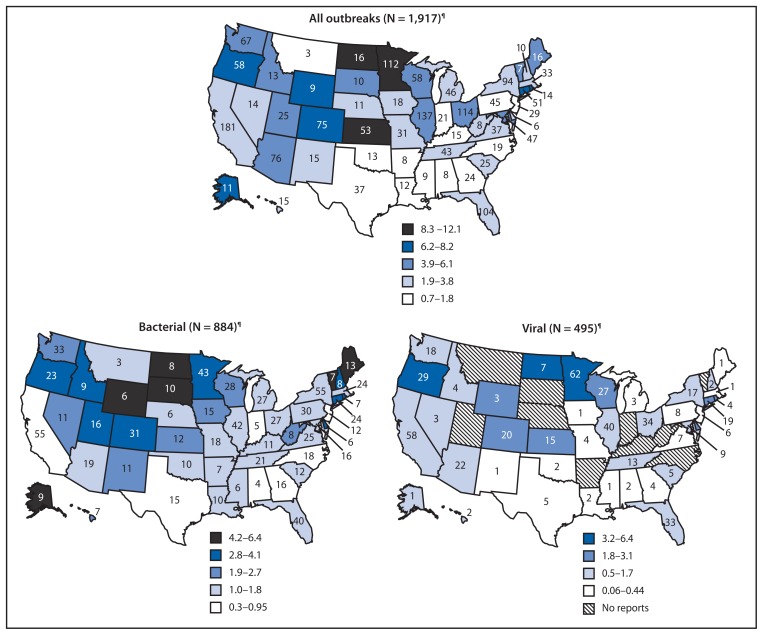
Average annual rate of reported foodborne disease outbreaks per 1 million population* and number of outbreaks,^†^ by state and major etiology group^§^ — Foodborne Disease Outbreak Surveillance System, United States, 2009–2010 * Cutpoints for outbreak rate categories determined using Jenks Natural Breaks Optimization in ArcGIS. Legend differs for each major etiology. ^†^ Number of reported outbreaks in each state. In addition to the 50 states, Puerto Rico reported 10 outbreaks, and the District of Columbia reported four outbreaks. ^§^ Analysis restricted to outbreaks with a single confirmed or suspected etiology. ^¶^ Includes 38 multistate outbreaks (i.e., outbreaks in which exposure to the etiologic agent occurred in more than one state) assigned as an outbreak to each state involved. Multistate outbreaks involved a median of seven (range: 2–45) states.

**TABLE 1:** Number and percentage of reported foodborne disease outbreaks, outbreak-associated illnesses, and hospitalizations, by etiology (confirmed or suspected)* — Foodborne Disease Outbreak Surveillance System, 2009–2010

Etiology	Outbreaks						Illnesses						Hospitalizations					
		
	2009		2010		Total 2009 and 2010		2009		2010		Total 2009 and 2010		2009		2010		Total 2009 and 2010	
								
	CE	SE	CE	SE	No.	(%)	CE	SE	CE	SE	No.	(%)	CE	SE	CE	SE	No	(%)
**Bacterial**																		
*Salmonella*†	113	4	121	5	243	(24)	2,924	30	2,274	1,861	7,089	(24)	286	4	291	2	583	(49)
*Clostridium perfringens*	19	7	17	14	57	(6)	964	866	1,062	333	3,225	(11)	0	0	9	0	9	(1)
*Escherichia coli,* Shiga toxin–producing (STEC)§	34	1	24	1	60	(6)	439	2	203	7	651	(2)	113	2	72	3	190	(16)
*Campylobacter*¶	13	2	21	4	40	(4)	292	8	259	41	600	(2)	10	1	9	2	22	(2)
Bacillus**	5	6	7	7	25	(2)	179	41	178	29	427	(2)	0	1	2	0	3	0
*Staphylococcus enterotoxin*††	4	7	5	3	19	(2)	95	29	118	10	252	(1)	0	0	0	0	0	0
*Shigella*§§	3	0	5	0	8	(1)	123	0	385	0	508	(2)	3	0	16	0	19	(2)
*Clostridium botulinum*	1	0	2	0	3	0	2	0	4	0	6	0	2	0	2	0	4	0
Other bacterial	0	2	0	0	2	0	0	174	0	0	174	(1)	0	0	0	0	0	0
*Listeria*¶¶	2	0	7	0	9	(1)	10	0	39	0	49	0	5	0	35	0	40	(3)
*Vibrio parahaemolyticus*	2	0	2	3	7	(1)	18	0	7	8	33	0	1	0	2	0	3	0
*Vibrio* other	0	0	1	0	1	0	0	0	4	0	4	0	0	0	2	0	2	0
*Enterococcus faecalis*	1	0	0	0	1	0	13	0	0	0	13	0	0	0	0	0	0	0
*Escherichia coli,* Enteropathogenic	0	0	1	0	1	0	0	0	7	0	7	0	0	0	4	0	4	0
*Escherichia coli,* Enterotoxigenic	2	0	0	1	3	0	66	0	0	19	85	0	0	0	0	0	0	0
*Brucella sp.*	1	0	0	0	1	0	4	0	0	0	4	0	1	0	0	0	1	0
**Total**	**200**	**29**	**213**	**38**	**480**	**(47)**	**5,129**	**1,150**	**4,540**	**2,308**	**13,127**	**(45)**	**421**	**8**	**444**	**7**	**880**	**(74)**
**Chemical and toxin**																		
Scombroid toxin/Histamine	7	0	11	0	18	(2)	32	0	44	0	76	0	0	0	0	0	0	0
Ciguatoxin	10	0	5	0	15	(1)	36	0	25	0	61	0	3	0	3	0	6	(1)
Other chemical	1	1	0	1	3	0	6	3	0	2	11	0	0	0	0	1	1	0
Mycotoxins	1	0	0	1	2	0	2	0	0	6	8	0	0	0	0	0	0	0
Paralytic shellfish poison	0	0	1	0	1	0	0	0	3	0	3	0	0	0	2	0	2	0
Pesticides	1	0	1	0	2	0	39	0	3	0	42	0	1	0	1	0	2	0
Plant/Herbal toxins	1	0	0	0	1	0	6	0	0	0	6	0	1	0	0	0	1	0
Other natural toxins	0	0	0	1	1	0	0	0	0	2	2	0	0	0	0	0	0	0
**Total**	**21**	**1**	**18**	**3**	**43**	**(4)**	**121**	**3**	**75**	**10**	**209**	**(1)**	**5**	**0**	**6**	**1**	**12**	**(1)**
**Parasitic**																		
*Cyclospora*	1	0	0	0	1	0	8	0	0	0	8	0	0	0	0	0	0	0
*Giardia lamblia*	0	0	1	0	1	0	0	0	5	0	5	0	0	0	1	0	1	0
**Total**	**1**	**0**	**1**	**0**	**2**	**0**	**8**	**0**	**5**	**0**	**13**	**0**	**0**	**0**	**1**	**0**	**1**	**0**
**Viral**																		
Norovirus	129	66	202	94	491	(48)	2948	968	4384	1437	9737	(33)	28	13	51	17	109	(9)
Hepatitis A	2	0	2	0	4	0	40	0	7	0	47	0	14	0	5	0	19	(2)
Rotavirus	0	1	0	0	1	0	0	28	0	0	28	0	0	1	0	0	1	0
Other viral	0	0	1	0	1	0	0	0	13	0	13	0	0	0	0	0	0	0
**Total**	**131**	**67**	**205**	**94**	**497**	**(49)**	**2,988**	**996**	**4,404**	**1,437**	**9,825**	**(33)**	**42**	**14**	**56**	**17**	**129**	**(11)**
**Known etiology** ***	353	97	437	135	1,022	(66)	8,246	2,149	9,024	3,755	23,174	(78)	468	22	507	25	1022	(86)
**Unknown etiology** †††	0	212	0	263	475	(31)	0	3,013	0	2,441	5,454	(19)	0	34	0	65	99	(8)
**Multiple etiologies**	11	2	14	3	30	(2)	287	64	408	57	816	(3)	29	1	32	1	63	(5)
**Total (all etiologies)** §§§	**364**	**311**	**451**	**401**	**1,527**	**(99)**	**8,533**	**5,226**	**9,432**	**6,253**	**29,444**	**(100)**	**497**	**57**	**539**	**91**	**1,184**	**(100)**

**Abbreviations:** CE = confirmed etiology; SE = suspected etiology.

* If at least one etiology was laboratory-confirmed, the outbreak was considered to have a confirmed etiology. If no etiology was laboratory-confirmed, but an etiology was reported based on clinical or epidemiologic features, the outbreak was considered to have a suspected etiology.

† Salmonella serotypes causing more than five outbreaks are Enteriditis (76 outbreaks), Newport (29), Typhimurium (27), Heidelberg (15), Montevideo (nine), Javiana (eight), Oranienburg (seven), Saintpaul (six) and Infantis (six).

§ STEC O111 (one confirmed outbreak), STEC O121:H19 (one confirmed outbreak), O145 (one confirmed outbreak) STEC O157:H7 (53 confirmed outbreaks), O26 (one confirmed outbreak), O26:H11 (one confirmed outbreak).

¶ Campylobacter jejuni (31 confirmed outbreaks, four suspected outbreaks), Campylobacter unknown (three confirmed outbreaks, two suspected outbreaks).

** Bacillus cereus (12 confirmed outbreaks, 12 suspected outbreaks), Bacillus unknown (one suspected outbreak).

†† Staphylococcus aureus (nine confirmed outbreaks, 10 suspected outbreaks).

§§ Shigella sonnei (eight confirmed outbreaks).

¶¶ Listeria monocytogenes (nine confirmed outbreaks).

*** The denominator for the total etiology percentages is the known etiology total. The denominator for the known etiology, unknown etiology, and multiple etiologies percentages is the total (all etiologies).

††† An etiologic agent was not confirmed or suspected based on clinical, laboratory, or epidemiologic information.

§§§ Because of rounding, numbers might not add up to the etiology category total or the known etiology total.

**TABLE 2:** Number of reported foodborne disease outbreaks and outbreak-associated illnesses, by etiology (confirmed or suspected)* and food commodity status — United States, 2009–2010

Etiology	No. of outbreaks (No. of illnesses)							

	Attributed to a single commodity		Attributed to food vehicle containing >1 commodity		Attributed to unknown commodity		Total	
**Bacterial**								
*Salmonella*†	71	(4,210)	44	(914)	128	(1,965)	**243**	**(7,089)**
*Clostridium perfringens*	24	(853)	23	(1,078)	10	(1,294)	**57**	**(3,225)**
*Escherichia coli,* Shiga toxin–producing (STEC)§	29	(363)	12	(145)	19	(143)	**60**	**(651)**
*Campylobacter*¶	22	(380)	2	(30)	16	(190)	**40**	**(600)**
Bacillus**	12	(206)	9	(201)	4	(20)	**25**	**(427)**
*Staphylococcus enterotoxin*††	3	(22)	12	(219)	4	(11)	**19**	**(252)**
*Shigella*§§	1	(96)	2	(329)	5	(83)	**8**	**(508)**
*Clostridium botulinum*	3	(6)	0	0	0	0	**3**	**(6)**
Other bacterial	0	0	1	(167)	1	(7)	**2**	**(174)**
*Listeria*¶¶	5	(25)	2	(10)	2	(14)	**9**	**(49)**
*Vibrio parahaemolyticus*	6	(30)	0	0	1	(3)	**7**	**(33)**
*Vibrio* other	1	(4)	0	0	0	0	**1**	**(4)**
*Enterococcus faecalis*	0	0	1	(13)	0	0	**1**	**(13)**
*Escherichia coli*, Enteropathogenic	0	0	0	0	1	(7)	**1**	**(7)**
*Escherichia coli*, Enterotoxigenic	0	0	2	(77)	1	(8)	**3**	**(85)**
*Brucella sp.*	1	(4)	0	0	0	0	**1**	**(4)**
**Total**	**178**	**(6,199)**	**110**	**(3,183)**	**192**	**(3,745)**	**480**	**(13,127)**
**Chemical and toxin**								
Scombroid toxin/Histamine	12	(55)	4	(13)	2	(8)	**18**	**(76)**
Ciguatoxin	12	(49)	0	0	3	(12)	**15**	**(61)**
Other chemical	1	(2)	1	(6)	1	(3)	**3**	**(11)**
Mycotoxins	1	(6)	1	(2)	0	0	**2**	**(8)**
Paralytic shellfish poison	1	(3)	0	0	0	0	**1**	**(3)**
Pesticides	0	0	2	(42)	0	0	**2**	**(42)**
Plant/Herbal toxins	1	(6)	0	0	0	0	**1**	**(6)**
Other natural toxins	1	(2)	0	0	0	0	**1**	**(2)**
**Total**	**29**	**(123)**	**8**	**(63)**	**6**	**(23)**	**43**	**(209)**
**Parasitic**								
*Cyclospora*	1	(8)	0	0	0	0	**1**	**(8)**
*Giardia lamblia*	0	0	0	0	1	(5)	**1**	**(5)**
**Total**	**1**	**(8)**	**0**	**0**	**1**	**(5)**	**2**	**(13)**
**Viral**								
Norovirus	40	(778)	135	(3,254)	316	(5,705)	**491**	**(9,737)**
Hepatitis A	1	(5)	1	(2)	2	(40)	**4**	**(47)**
Rotavirus	0	0	0	0	1	(28)	**1**	**(28)**
Other viral	0	0	0	0	1	(13)	**1**	**(13)**
**Total**	**41**	**(783)**	**136**	**(3,256)**	**320**	**(5,786)**	**497**	**(9,825)**
**Known etiology** ***	249	(7,113)	254	(6,502)	519	(9,559)	**1,022**	**(23,174)**
**Unknown etiology** †††	41	(815)	74	(982)	360	(3657)	**475**	**(5454)**
**Multiple etiologies**	9	(264)	8	(232)	13	(320)	**30**	**(816)**
**Total (all etiologies)** §§§	**299**	**(8,192)**	**336**	**(7,716)**	**892**	**(13,536)**	**1,527**	**(29,444)**

* If at least one etiology was laboratory-confirmed, the outbreak was considered to have a confirmed etiology. If no etiology was lab-confirmed, but an etiology was reported based on clinical or epidemiologic features, the outbreak was considered to have a suspected etiology.

† Salmonella serotypes causing more than five outbreaks are Enteriditis (76 outbreaks), Newport (29), Typhimurium (27), Heidelberg (15), Montevideo (nine), Javiana (eight), Oranienburg (seven), Saintpaul (six) and Infantis (six).

§ STEC O111 (one confirmed outbreak), STEC O121:H19 (one confirmed outbreak), O145 (one confirmed outbreak) STEC O157:H7 (53 confirmed outbreaks), O26 (one confirmed outbreak), O26:H11 (1 confirmed outbreak).

¶ Campylobacter jejuni (31 confirmed outbreaks, four suspected outbreaks), Campylobacter unknown (three confirmed outbreaks, two suspected outbreaks).

** Bacillus cereus (12 confirmed outbreaks, 12 suspected outbreaks), Bacillus unknown (one suspected outbreak).

†† Staphylococcus aureus (nine confirmed outbreaks, 10 suspected outbreaks).

§§ Shigella sonnei (eight confirmed outbreaks).

¶¶ Listeria monocytogenes (nine confirmed outbreaks).

*** The denominator for the total etiology percentages is the known etiology total. The denominator for the known etiology, unknown etiology, and multiple etiologies percentages is total (all etiologies).

††† An etiologic agent was not confirmed or suspected based on clinical, laboratory, or epidemiologic information.

§§§ Because of rounding, numbers might not add up to the etiology category total or the known etiology total.

## Footnotes

* The reporting form and guidance for reporting via the National Outbreak Reporting System are available at http://www.cdc.gov/nors.

† If at least one etiology was laboratory-confirmed, the outbreak was considered to have a confirmed etiology. If no etiology was laboratory-confirmed, but an etiology was reported based on clinical or epidemiologic features, the outbreak was considered to have a suspected etiology. A guide to confirming foodborne disease diagnoses in outbreaks is available at http://www.cdc.gov/outbreaknet/references_resources/guide_confirming_diagnosis.html.

§ The 17 mutually exclusive food commodities are as follows: fish, crustaceans, mollusks, dairy, eggs, beef, game, pork, poultry, grains-beans, oils-sugars, fruits-nuts, fungi, sprouts, and leafy, root, and vine-stalk vegetables.

¶ Available at http://www.census.gov/popest.

** Defined as those vegetables that grow on either a vine or a stalk, such as tomatoes, corn, cucumbers, eggplant, green beans, peppers, pumpkin, zucchini, okra, peas, and squash.

†† Additional information on product recalls is available at http://www.fda.gov/safety/recalls/default.htm and http://www.fsis.usda.gov/fsis_recalls/index.asp.

§§ Available at http://www.nasda.org/file.aspx?id=3916.

## References

[1] Scallan E, Hoekstra RM, Angulo FJ (2011). Foodborne illness acquired in the United States—major pathogens. Emerg Infect Dis.

[2] Painter JA, Ayers T, Woodruff R (2009). Recipes for foodborne outbreaks: a scheme for categorizing and grouping implicated foods. Foodborne Pathog Dis.

[3] Neil KP, Biggerstaff G, MacDonald JK (2012). A novel vehicle for transmission of *Escherichia coli* O157:H7 to humans: multistate outbreak of *E. coli* O157:H7 infections associated with consumption of ready-to-bake commercial prepackaged cookie dough—United States, 2009. Clin Infect Dis.

[4] CDC (2011). Surveillance for foodborne disease outbreaks—United States, 2008. MMWR.

[5] CDC (2011). Updated norovirus outbreak management and disease prevention guidelines. MMWR.

[6] Hall AJ, Eisenbart VG, Etingue AL, Gould LH, Lopman B, Parashar UD (2012). Epidemiology of foodborne norovirus outbreaks, United States, 2001–2008. Emerg Infect Dis.

[7] American Public Health Association (2006). The public health workforce shortage: left unchecked, will we be protected?.

[8] National Association of County and City Health Officials (2012). Local health department job losses and program cuts: findings from January/February 2010 survey.

[9] US Department of Health and Human Services (2000). Food safety. Healthy people 2010 (midcourse review).

[10] Langer AJ, Ayers T, Grass J, Lynch M, Angulo FJ, Mahon BE (2012). Nonpasteurized dairy products, disease outbreaks, and state laws—United States, 1993–2006. Emerg Infect Dis.

